# Efficacy and safety of Xiaoaiping injection for breast cancer: A protocol for systematic review and meta-analysis

**DOI:** 10.1097/MD.0000000000021932

**Published:** 2020-08-28

**Authors:** Daorui Hou, Jian Xiong, Yahui Peng, Ya Li, Lu Xiong

**Affiliations:** a Department of Traditional Chinese Medicine Oncology, The First People's Hospital of Xiangtan City, Xiangtan, Hunan Province; b Department of Oncology, Guang’anmen Hospital; c Beijing Shahe Hospital, Beijing; d Hangzhou Lin’an TCM Hospital, Hangzhou, Zhejiang Province, China.

**Keywords:** breast cancer, protocol, systematic review and meta-analysis, Xiaoaiping

## Abstract

**Background:**

Xiaoaiping injection, extracted from the Chinese herb Marsdenia tenacissima (Roxb.) Wight et Arn., is a broad-spectrum anti-tumor drug and has been widely used for the treatment of breast cancer in China. The aim of this study is to systematically investigate the efficacy and safety of Xiaoaiping injection for the treatment of breast cancer.

**Methods:**

We will perform the comprehensive literature search in the following databases from their inceptions to August 2020 for data extraction: PubMed, the Cochrane Library, Embase, the China National Knowledge Infrastructure, Wanfang Database, Chinese Science and Technology Periodical Database, and Chinese Biomedical Literature Database. Cochrane Risk of Bias tool will be used to assess the risk of bias of included studies. The RevMan 5.4 and Stata 16.0 software will be applied for statistical analyses. Statistical heterogeneity will be computed by I2 tests. Sensitivity analysis will be conducted to evaluate the stability of the results. The publication bias will be evaluated by funnel plots and Egger's test. The quality of evidence will be assessed by the GRADE system.

**Results:**

The results of our research will be published in a peer-reviewed journal.

**Conclusion:**

The conclusion of this study will provide evidence to show whether Xiaoaiping injection is an effective intervention for patient with breast cancer.

**OSF registration number:**

10.17605/OSF.IO/4ZUXC

## 1 Introduction

Breast cancer, ranking as the second-leading cause of cancer-related deaths in women, is one of the most common malignancy.^[1,2]^ In the past few years, the breast cancer incidence rate increased slightly by 0.3% per year in America.^[3]^ In recent years, therapies for breast cancer have evolved rapidly, from chemotherapy and radiotherapy to targeted therapy and immunotherapy.^[4,5]^ However, these therapies also experience intractable issues, such as acquired resistance, low tumor response rates, and unaffordable medical expenses.^[6]^

Traditional Chinese medicine (TCM) has been effectively applied in treating malignant diseases for thousands of years in Eastern Asia.^[7–9]^ Researches have shown that Chinese medicine combined with chemotherapy can significantly enhance tumor response and alleviate toxicity compared with chemotherapy alone.^[10]^ Xiaoaiping injection, mainly composed of the Chinese herb *Marsdenia tenacissima (Roxb.) Wight et Arn*., was approved by the China Food and Drug Administration (CFDA) for tumor treatment with approval number Z20025868. It has exhibited antitumor effects on various cancers such as ovarian cancer,^[11,12]^ esophageal cancer,^[13]^ gastric cancer,^[14]^ lung cancer,^[15,16]^ liver cancer,^[17,18]^ and breast cancer.^[19,20]^

Studies have shown that Xiaoaiping injection can significantly reverse multidrug resistance of cancer,^[21]^ enhance efficacies of chemotherapy,^[20,22]^ and reduce the side effects induced by chemotherapy in breast cancer patients.^[19]^ However, there is still a lack of high-quality evidence to support the effectiveness and safety of Xiaoaiping injection on patients with breast cancer. In this work, we will perform a systematic review to evaluate the effificacy and safety of Xiaoaiping injection in the treatment of breast cancer and to provide a reference for clinical application.

## 2 Methods and analysis

This study was prospectively registered in the Open Science Framework (OSF) with a DOI: 10.17605/OSF.IO/4ZUXC. It will be carried out under the guideline of Preferred Reporting Items for Systematic Reviews and Meta-analyses Protocols.^[23]^

### 2.1 Inclusion criteria

#### 2.1.1 Type of study

All randomized controlled trials (RCTs) of Xiaoaiping injection for the treatment of breast cancer will be included without language restriction. Observational studies, cross-over studies, conference abstracts, animal studies, and letters will be excluded.

#### 2.1.2 Types of participants

We will include RCTs on participants who are diagnosed as breast cancer. There are no limits to research subjects age, sex, race, condition duration, or intensity.

#### 2.1.3 Types of interventions

Interventions to be reviewed are Xiaoaiping injection alone or combinations with other interventions to treat the breast cancer. When Xiaoaiping injection used as combinations with other treatments, the control group should also receive the same combination treatments.

#### 2.1.4 Types of outcomes

The primary outcome indicators involve overall survival (OS) and progression-free survival (PFS). Overall response rate (ORR), disease control rate (DCR), quality of life improved rate (QIR), and adverse events (AEs) will be regarded as the secondary outcome indicators.

### 2.2 Search strategy

Two researchers will independently retrieve the following databases from their inceptions to August 2020: PubMed, the Cochrane Library, Embase, the China National Knowledge Infrastructure, Wanfang Database, Chinese Science and Technology Periodical Database, and Chinese Biomedical Literature Database. Google scholar, Bing scholar, and Baidu scholar will also be retrieved to find out other related literature. In addition, we will identify grey literatures to avoid missing any potential studies, such as dissertations, ongoing trials from clinical trials registries, conference abstracts, and reference lists of associated reviews. An example of search strategy for PubMed database that combines MeSH terms and free words will be adopted. The search strategy was as follows:

#1 Search: (“Breast Neoplasms”[Mesh]) OR (((((((((((((((((((((((((((((((((((((Breast Neoplasm[Title/Abstract]) OR (Neoplasm, Breast[Title/Abstract])) OR (Breast Tumors[Title/Abstract])) OR (Breast Tumor[Title/Abstract])) OR (Tumor, Breast[Title/Abstract])) OR (Tumors, Breast[Title/Abstract])) OR (Neoplasms, Breast[Title/Abstract])) OR (Breast Cancer[Title/Abstract])) OR (Cancer, Breast[Title/Abstract])) OR (Mammary Cancer[Title/Abstract])) OR (Cancer, Mammary[Title/Abstract])) OR (Cancers, Mammary[Title/Abstract])) OR (Mammary Cancers[Title/Abstract])) OR (Malignant Neoplasm of Breast[Title/Abstract])) OR (Breast Malignant Neoplasm[Title/Abstract])) OR (Breast Malignant Neoplasms[Title/Abstract])) OR (Malignant Tumor of Breast[Title/Abstract])) OR (Breast Malignant Tumor[Title/Abstract])) OR (Breast Malignant Tumors[Title/Abstract])) OR (Cancer of Breast[Title/Abstract])) OR (Cancer of the Breast[Title/Abstract])) OR (Mammary Carcinoma, Human[Title/Abstract])) OR (Carcinoma, Human Mammary[Title/Abstract])) OR (Carcinomas, Human Mammary[Title/Abstract])) OR (Human Mammary Carcinomas[Title/Abstract])) OR (Mammary Carcinomas, Human[Title/Abstract])) OR (Human Mammary Carcinoma[Title/Abstract])) OR (Mammary Neoplasms, Human[Title/Abstract])) OR (Human Mammary Neoplasm[Title/Abstract])) OR (Human Mammary Neoplasms[Title/Abstract])) OR (Neoplasm, Human Mammary[Title/Abstract])) OR (Neoplasms, Human Mammary[Title/Abstract])) OR (Mammary Neoplasm, Human[Title/Abstract])) OR (Breast Carcinoma[Title/Abstract])) OR (Breast Carcinomas[Title/Abstract])) OR (Carcinoma, Breast[Title/Abstract])) OR (Carcinomas, Breast[Title/Abstract]))
#2 Search: (“Marsdeniae tenacissimae extract” [Supplementary Concept]) OR (((((((Xiao-Ai-Ping[Title/Abstract]) OR (Xiaoaiping[Title/Abstract])) OR (XAP[Title/Abstract])) OR (XAPI[Title/Abstract])) OR (Marsdenia tenacissima[Title/Abstract])) OR (MTE[Title/Abstract])) OR (tongguanteng[Title/Abstract]))
#3 Search: (((((((((randomized controlled trial[Title/Abstract]) OR RCT[Title/Abstract]) OR random[Title/Abstract]) OR randomly[Title/Abstract]) OR random allocation[Title/Abstract]) OR allocation[Title/Abstract]) OR randomized control trial[Title/Abstract]) OR controlled clinical trial[Title/Abstract]) OR clinical trial[Title/Abstract]) OR clinical study[Title/Abstract]
#1 and #2 and #3

### 2.3 Study selection and data extraction

#### 2.3.1 Selection of studies

**Figure 1:**
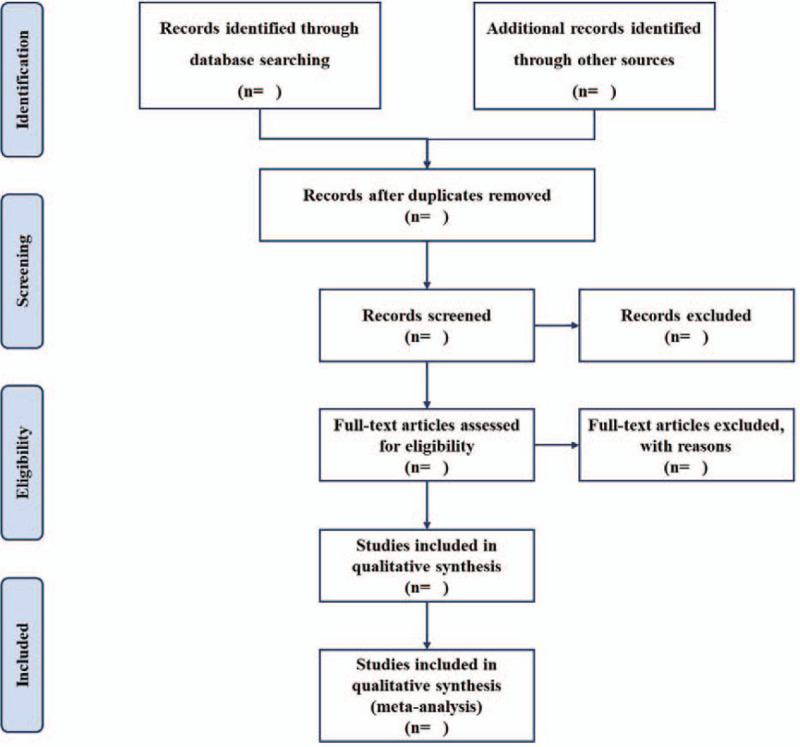
Flow chart of study selection.

The electronic citations extracted out from the above databases will be managed by EndNote X9.0 (Thomson Corporation, Connecticut).^[24]^ The titles and abstracts of all searched studies will be assessed independently by 2 methodological trained authors in accordance with the established selection criteria. Full papers of potential studies will be reviewed if necessary. Any divergences between 2 authors will be solved through discussion with a third author. Excluded studies will be listed in a table with reasons. A Preferred Reporting Items for Systematic Reviews and Meta-analysis flow chart (Fig. 1) will be drawn to present the whole process of study selection.

#### 2.3.2 Data extraction and management

Two authors will independently extract relevant data with the standardized sheet recommended by the Cochrane Handbook of Systematic Reviews of Interventions. The data of those qualified articles will be export to Microsoft Excel, which includes basic information (registered identification, first author, author's unit, country, and publication year), research design (sample size, random sequence generation, allocation concealment, analysis of the data, processing of missing data, blinding of the participants, blinding of the outcome measurement, and blinding of the assessors), participants (disease, age, disease stage, and diagnostic criteria), details of treatment and comparison (e.g., delivery methods, dosage, and frequency), outcomes (outcome measurement), adverse events, conflicts of interest, and other essential information. If unclear or missing data are examined, we will contact primary authors to achieve it whenever possible. Any unresolved disagreements between 2 authors will be solved through discussion with another senior author.

#### 2.3.3 Assessment of risk of bias

A tool introduced in the Cochrane Handbook for Systematic Reviews of Interventions will be used to assess a broad category of biases. In this tool, the risk of bias of a trial is evaluated through 7 items, include random sequence generation, allocation concealment, blinding of the participants and personnel, blinding of the outcome assessments, incomplete outcome data, selective reporting, and other sources of bias. The bias risk assessment is divided into 3 criteria: “Low risk,” “High risk,” or “Unclear risk.” Inconsistencies will be resolved by discussion within the group.

#### 2.3.4 Synthesis of data

A meta-analysis will be carried through using RevMan 5.4 (The Cochrane Collaboration, Oxford, England) and Stata 16.0 software (Stata 16.0, College Station, TX). Dichotomous data will be reported as risk ratio (RR) with 95% confidence interval (CI), whereas continuous data will be reported as mean difference (MD) or standard mean difference (SMD) with 95% CI. MD will be used when the treatment outcome was measured by the same scale. SMD will be used when the treatment outcome was measured by different scales in different studies.

#### 2.3.5 Assessment of heterogeneity

Statistical heterogeneity will be identified by *I*^2^ statistics.^[25]^ Acceptable heterogeneity is considered if *I*^2^ ≤ 50% and a fixed-effect model will be applied. Otherwise, obvious heterogeneity is regarded if *I*^2^ > 50%, and a random-effect model will be utilized.

#### 2.3.6 Subgroup analysis

In the case of high heterogeneity, we will conduct subgroup analysis according to the region of the studies, age, stage of the subjects, types of treatments, and different outcomes. We will evaluate the credibility of the subgroup analysis in term of the guidance.^[26]^ If quantitative synthesis is not appropriate due to substantial heterogeneity, then systematic review will be conducted and the results will be displayed in tables and figures.

#### 2.3.7 Sensitivity analysis

Sensitivity analysis will be conducted to identify the robustness of the result and detect whether there are any exceptional studies bringing about an evident heterogeneity. We will exclude each study included in the analysis one by one. Then we will reanalyze and compile the data and compare the difference between the reobtained effects and the original effects. If there is one or more very large study, we will repeat the analysis excluding them to determine how much they dominate the results.

#### 2.3.8 Assessment of reporting bias

When there are sufficient studies available (normally over 10 studies), we will check the reporting bias using funnel plot and Egger regression test.^[27,28]^*P* < .05 is considered to have publication bias.

#### 2.3.9 Grading the quality of evidence

We will assess the quality of evidence using The Grading of Recommendations Assessment, Development and Evaluation (GRADE), a widely used tool in evaluating the quality of assessment.^[29]^ The quality of evidence will be rated as high, moderate, low, or very low.

### 2.4 Patient and public involvement

Patient and public were not involved in this study.

### 2.5 Ethics and dissemination

This systematic review will not require ethical approval because there are no data used in our study that are linked to individual patient data. This systematic review will be disseminated through a peer-reviewed publication.

## 3 Discussion

Breast cancer is the most common malignancy in women. Xiaoaiping injection is a famous Chinese patent medicine for the treatment of breast cancer in clinical practice, and a series of clinical studies have been conducted on it.^[19,20]^ However, no systematic review related to Xiaoaiping injection for breast cancer has been published currently. In this study, we will conduct systematic review and meta-analysis to provide more evidence on the effectiveness and safety for it. These findings may provide helpful guidance for clinicians in the treatment of breast cancer.

### 3.1 Amendments

If amendments are needed, we will update our protocol to include any changes in the whole process of research.

## Author contributions

**Conceptualization:** Daorui Hou, Lu Xiong.

**Data curation:** Daorui Hou, Jian Xiong, Yahui Peng.

**Formal analysis:** Yahui Peng, Ya Li.

**Funding acquisition:** Lu Xiong.

**Investigation:** Jian Xiong, Yahui Peng, Ya Li.

**Methodology:** Daorui Hou, Jian Xiong, Lu Xiong.

**Project administration:** Lu Xiong.

**Resources:** Daorui Hou, Jian Xiong, Yahui Peng.

**Software:** Daorui Hou, Jian Xiong, Ya Li.

**Supervision:** Lu Xiong.

**Writing – original draft:** Daorui Hou, Jian Xiong.

**Writing – review & editing:** Daorui Hou, Jian Xiong, Yahui Peng, Ya Li, Lu Xiong.

## References

[1] Bray F Ferlay J Soerjomataram I Global cancer statistics 2018: GLOBOCAN estimates of incidence and mortality worldwide for 36 cancers in 185 countries. CA Cancer J Clin 2018;68:394 424. 30207593 10.3322/caac.21492

[2] Harbeck N Gnant M Breast cancer. Lancet 2017;389:1134 50. 27865536 10.1016/S0140-6736(16)31891-8

[3] DeSantis CE Ma J Gaudet MM Breast cancer statistics, 2019. CA Cancer J Clin 2019;69:438 51. 31577379 10.3322/caac.21583

[4] Arruebo M Vilaboa N Sáez-Gutierrez B Assessment of the evolution of cancer treatment therapies. Cancers (Basel) 2011;3:3279 330. 24212956 10.3390/cancers3033279 PMC3759197

[5] Falzone L Salomone S Libra M Evolution of cancer pharmacological treatments at the turn of the third millennium. Front Pharmacol 2018;9:1 26. 30483135 10.3389/fphar.2018.01300 PMC6243123

[6] D’Abreo N Adams S Immune-checkpoint inhibition for metastatic triple-negative breast cancer: safety first? Nat Rev Clin Oncol 2019;16:399 400. 31053774 10.1038/s41571-019-0216-2

[7] Hsiao WL Liu L The role of traditional Chinese herbal medicines in cancer therapy--from TCM theory to mechanistic insights. Planta Med 2010;76:1118 31. 20635308 10.1055/s-0030-1250186

[8] Xiang Y Guo Z Zhu P Traditional Chinese medicine as a cancer treatment: modern perspectives of ancient but advanced science. Cancer Med 2019;8:1958 75. 30945475 10.1002/cam4.2108 PMC6536969

[9] So TH Chan SK Lee VH Chinese medicine in cancer treatment - how is it practised in the East and the West? Clin Oncol 2019;31:578 88. 10.1016/j.clon.2019.05.016 31178347

[10] Zhu L Li L Li Y Chinese herbal medicine as an adjunctive therapy for breast cancer: a systematic review and meta-analysis. Evid Based Complement Alternat Med 2016;2016:1 7. 10.1155/2016/9469276 PMC4876224 27239216

[11] Zheng AW Chen YQ Fang J Xiaoaiping combined with cisplatin can inhibit proliferation and invasion and induce cell cycle arrest and apoptosis in human ovarian cancer cell lines. Biomed Pharmacother 2017;89:1172 7. 28320083 10.1016/j.biopha.2017.03.012

[12] Zhang XQ Ding YW Chen JJ Xiaoaiping injection enhances paclitaxel efficacy in ovarian cancer via pregnane X receptor and its downstream molecules. J Ethnopharmacol 2020;261:1 20. 10.1016/j.jep.2020.113067 32505840

[13] Wang F Fan QX Wang HH Efficacy and safety of Xiaoaiping combined with chemotherapy in the treatment of advanced esophageal cancer. Zhonghua Zhong Liu Za Zhi 2017;39:453 7. 28635236 10.3760/cma.j.issn.0253-3766.2017.06.010

[14] Zhou X Liu M Ren Q Oral and injectable Marsdenia tenacissima extract (MTE) as adjuvant therapy to chemotherapy for gastric cancer: a systematic review. BMC Complement Altern Med 2019;19:1 4. 31830977 10.1186/s12906-019-2779-y PMC6909592

[15] Han SY Zhao MB Zhuang GB Marsdenia tenacissima extract restored gefitinib sensitivity in resistant non-small cell lung cancer cells. Lung Cancer 2012;75:30 7. 21757251 10.1016/j.lungcan.2011.06.001

[16] Feng F Huang J Wang Z Xiao-ai-ping injection adjunct with platinum-based chemotherapy for advanced non-small-cell lung cancer: a systematic review and meta-analysis. BMC Complement Med Ther 2020;20:1 5. 32020869 10.1186/s12906-019-2795-y PMC7076846

[17] Zhan J Shi LL Wang Y In Vivo study on the effects of xiaoaiping on the stemness of hepatocellular carcinoma cells. Evid Based Complement Alternat Med 2019;2019:1 0. 10.1155/2019/4738243 PMC6612394 31341493

[18] Huang Z Wang Y Chen J Effect of Xiaoaiping injection on advanced hepatocellular carcinoma in patients. J Tradit Chin Med 2013;33:34 8. 23596809 10.1016/s0254-6272(13)60097-7

[19] Yu F Li Y Zou J The Chinese herb Xiaoaiping protects against breast cancer chemotherapy-induced alopecia and other side effects: a randomized controlled trial. J Int Med Res 2019;47:2607 14. 31099281 10.1177/0300060519842781 PMC6567696

[20] Ruan LW Deng YC Study on effect of Xiaoaiping in enhancing efficacy of neoadjuvant chemotherapy for breast cancer and its mechanism. Zhongguo Zhong Yao Za Zhi 2015;40:749 52. 26137702

[21] To KKW Wu X Yin C Reversal of multidrug resistance by Marsdenia tenacissima and its main active ingredients polyoxypregnanes. J Ethnopharmacol 2017;203:110 9. 28363522 10.1016/j.jep.2017.03.051

[22] Chen J Zhang X Xiao X Xiao-Ai-Ping injection enhances effect of paclitaxel to suppress breast cancer proliferation and metastasis via activating transcription factor 3. Integr Cancer Ther 2020;19:1 1. 10.1177/1534735420906463 PMC7136938 32248718

[23] Moher D Shamseer L Clarke M Preferred reporting items for systematic review and meta-analysis protocols (PRISMA-P) 2015 statement. Syst Rev 2015;4:1 9. 25554246 10.1186/2046-4053-4-1 PMC4320440

[24] Bramer W Bain P Updating search strategies for systematic reviews using EndNote. J Med Libr Assoc 2017;105:285 9. 28670219 10.5195/jmla.2017.183 PMC5490709

[25] Higgins JP Thompson SG Quantifying heterogeneity in a meta-analysis. Stat Med 2002;21:1539 58. 12111919 10.1002/sim.1186

[26] Sun X Briel M Walter SD Is a subgroup effect believable? Updating criteria to evaluate the credibility of subgroup analyses. BMJ 2010;340:850 4. 10.1136/bmj.c117 20354011

[27] Sutton AJ Duval SJ Tweedie RL Empirical assessment of effect of publication bias on meta-analyses. BMJ 2000;320:1574 7. 10845965 10.1136/bmj.320.7249.1574 PMC27401

[28] Egger M Davey Smith G Schneider M Bias in meta-analysis detected by a simple, graphical test. BMJ 1997;315:629 34. 9310563 10.1136/bmj.315.7109.629 PMC2127453

[29] Atkins D Best D Briss PA Grading quality of evidence and strength of recommendations. BMJ 2004;328:1490 4. 15205295 10.1136/bmj.328.7454.1490 PMC428525

